# PBL and sustainable education: addressing the problem of isolation

**DOI:** 10.1007/s10459-019-09927-z

**Published:** 2019-10-09

**Authors:** Liesbeth Noordegraaf-Eelens, Julien Kloeg, Gera Noordzij

**Affiliations:** 1 Erasmus School of Philosophy, Erasmus University Rotterdam, Rotterdam, The Netherlands; 2 Codarts Rotterdam, Rotterdam, The Netherlands; 3 Rotterdam Arts and Sciences Lab, Rotterdam, The Netherlands; 4 Erasmus University College, Nieuwemarkt 1A, 3011 HP Rotterdam, The Netherlands; 5 Department of Psychology, Education and Child Studies, Erasmus University Rotterdam, Rotterdam, The Netherlands

**Keywords:** Problem-based learning, Problem of isolation, Sustainable education, Responsibility for shared world, Hannah Arendt

## Abstract

Problem-based learning (PBL) is an innovative educational approach that dates back to the 1960s. However, the twenty-first century goal of sustainable education poses a challenge to PBL, especially as it relates to isolation. Here we discuss the underlying issue of isolation in three respects. First, the information-processing model of PBL depends on generalized skills, whereas real life problem-solving skills involve context-bound cognitive processes. Second, in all models of PBL, the focus on knowledge acquisition for a specific problem improves performance but separates education from the world at large. Third, the existing culture of measurement strengthens the aforementioned isolating effects. In response, we introduce a conceptual approach based on Hannah Arendt’s technical notion of ‘world’. We make suggestions to meet the criteria of sustainable education by reconnecting PBL to our shared world, and emphasizing a responsibility for this shared world.

## Introduction

Here we take a critical look at problem-based learning (PBL) as it relates to sustainable education. We focus on three different models of PBL: the McMaster model, the Maastricht model, and the Danish model. We then discuss the problem of isolation that can be found, both implicitly and explicitly, in the three models. First, the information-processing view of PBL introduced by Howard Barrows (Servant-Miklos 2018) assumes education involves highly general skills that transcend differences between different domains. We criticize this model on the basis that education becomes isolated, such that it leaves context behind. Second, PBL often proceeds in a separate space that exists in isolation from the world at large. Third, this isolation is reinforced by a societal culture of measurement (Biesta 2015). Though we are certainly convinced of the value of PBL, we propose an ‘update’ to better face the twenty-first century challenge of *sustainable education*, where sustainability “implies the survival, the security, and beyond these, the well-being of a whole system, whether this is seen at local level, such as community, or at global level” (Sterling 2010, p. 512). In particular, we argue this is possible by instilling responsibility for a shared world into the curriculum.

## The history of PBL and its central clash of paradigms

Problem-based learning (PBL) has been one of the most successful educational innovations in higher education from the last 50 years. PBL first emerged during the late 1960s at McMaster University in Hamilton, Canada. Most books on teaching and learning credit Howard Barrows as the pioneer of PBL (e.g., Stefaniak 2017), but Barrows himself acknowledged Jim Anderson as the creator of PBL as a “defined teacher-learning method” (Barrows 2000, p. 257). Others argue “the real brains behind the successful inception and development of the first PBL programme was Bill Spaulding” (Servant 2016, p. 38). Indeed, Bill Spaulding started a committee in 1966 with the aim of assembling a new medical curriculum, collaborating with Jim Anderson, Fraser Mustard, Bill Walsh, and the founding Dean of McMaster School of Medicine, John Evans. Barrows joined this committee in 1968, and later he formalized the appellation ‘problem-based learning’.

This committee established three core principles of PBL: (1) a self-directed, small-group, problem-based approach; (2) a systems-based approach to the curriculum; and (3) a community-oriented attitude to ensure a link to larger society (Servant 2016). Though the first two are still upheld as core principles of PBL, the third did not survive the start of the medical curriculum at McMaster. That said, instigated by the World Health Organization, different medical schools established a network in 1979 to support institutions that wished to adapt their curriculum to the health needs of their community, and PBL was trumpeted as a tool to achieve this community-orientated education (Schmidt 1989). As will be discussed later, this focus on community can be found in the Danish model at the University of Alborg (De Graaf and Kolmos 2003).

When the new PBL-based curriculum was launched at McMaster in 1969, it immediately attracted broad international attention, especially from the newly founded medical faculty at Maastricht University in the Netherlands (Schmidt 2012). Aside from minor differences in curriculum structure and the number of students assigned to groups, the implementation of PBL and its rationale were remarkably similar between the McMaster and Maastricht models. Both institutions saw problems as the starting point of learning. Problems were likewise formulated by a teacher and followed clearly defined learning objectives. But beneath these similarities there are deep differences in the underlying philosophy and the interpretations of learning in PBL. The information-processing view advocated by Howard Barrows from McMaster University posits that PBL fosters problem-solving and reasoning skills. In contrast, the constructivist or knowledge acquisition view advocated by Henk Schmidt from Maastricht University posits that PBL helps students generate a ‘mental model’ underlying a problem (Servant-Miklos 2018).

Independently and in parallel to the development of PBL at McMaster and Maastricht, project learning in engineering education emerged in at the University of Alborg in Denmark. So-called problem-oriented projects are meant to foster critical thinking and community orientation by tackling societal problems in the curriculum (Kolmos 2017). Knud Illeris has been one pioneer of the Danish model of PBL (Hüttel and Gnaur 2017; Servant 2016). In 1974, Illeris formulated four principles for this educational model: problem-orientation, interdisciplinarity, project work in small groups, and experience-based learning. Those principles are still the main focus of the Danish model today (De Graaf and Kolmos 2003). So, whereas both the McMaster and the Maastricht model focus on a self-directed, small-group, problem-based approach to learning, and a systems-based approach to the curriculum, the community-oriented approach is unique to the Danish model.

PBL has now spread to over 500 higher education institutions all over the world, and is no longer limited to medical or engineering education, but is also used in economics and business, psychology, biology, and law (Schmidt et al. 2009). The application of PBL might be different among all these institutions, but it is widely acknowledged that PBL serves as a broad educational strategy to organize the learning process of students in such a way that they are actively engaged. All models are student-centered with the teacher acting as process facilitator; all take place in small groups; learning is organized around problems; and all models promote students’ self-directed learning and collaboration.

While we support these features of PBL, it is an open question whether the current models of PBL represent a sustainable form of education (Sterling 2001). Although initially designed by the first educational committee of McMaster to be “community-oriented”, many implementations of PBL hardly seem connected to society at large. Take for example a student in medicine. Within her PBL education, the focus will be on one PBL problem, either defined by the teacher or by the students. First of all, this does not resemble the actual situation a student will have to deal with after her studies are finished; a doctor will deal with several medical problems at the same time, as well as keep in mind work–life balance and time management. This goes beyond the immediate medical context, or even the organisational or larger personal context, and extends into the ‘shared world’ as we call it here. For instance, the global effects of antibiotic prescriptions have to be taken into account. The doctor’s decisions in response to such problems become part of the world that we humans create for ourselves and are constantly shaping (Arendt 1961, 1998). We claim that the responsibility for sustainability cannot be neglected by an educational programme that wishes to be community-oriented.

In the sections that follow, we develop what we call the issue of isolation. This issue arises in two different respects, and is strengthened by a third societal factor—namely, the existing culture of measurement.

## First critique: isolation through generalized skills

A key difference between the McMaster, Maastricht, and Danish models lies in the purpose and use of problems. In both the McMaster and Maastricht models, problems are written and defined by teachers and subject-matter experts, and act as the starting point for learning. In the Danish model, however, students first analyze and define the problem within a given domain or interdisciplinary context, then start their project. As a result, in the Danish model students’ degree of self-direction is higher, they learn more skills (e.g., planning, monitoring), and are able to apply their knowledge to real-life situations. Though it might seem like McMaster and Maastricht formulate problems in the same way, a closer look reveals they fundamentally differ on this point. This difference stems from the distinction between the information-processing model at the heart of the McMaster model, and the knowledge-acquisition model at the heart of the Maastricht model (Servant-Miklos 2018). McMaster no longer practices the information-processing model (Neville and Norman 2007), yet this model continues to inform many PBL curricula (Schmidt et al. 2009) and focuses on the development of generalized problem-solving skills with only minimal contextualization in terms of the specific problems addressed; for instance, general clinical reasoning skills as such are highly sought after (Barrows and Tamblyn 1980).

This idea of generalized skills depends on an underlying information-processing theory of psychology, heavily inspired by the computer science-based approaches of Newell and Simon (1972). They described problem-solving as a general skill, independent of the content of the problem. Methodologically speaking, the measurement of these generalized skills was found to be flawed (Ohlsson 2012), and theoretically speaking, problem-solving skills were shown to be context-bound (Servant-Miklos 2018). There is therefore limited evidence to support the ‘General Problem Solver’ as envisioned by Newell and Simon. Instead, there is a range of cognitive strategies that are made relevant by the specific context, for instance the content of a PBL problem. This relation between problem-solving and context is hard to incorporate within the information-processing model because it assumes the goal of PBL is the development of skills that are nearly devoid of content—i.e., independent of context—such as the aforementioned clinical reasoning skills. In this sense, the information-processing model incorrectly represents education as an isolated activity that leaves considerations of context behind. Education does not operate on this level of abstract generality. Rather, one of the central questions in education should be *how* to incorporate context to develop specific skills or mental models.

## Second critique: isolation and the separate space of education

The Maastricht model strives to offer a more contextualized notion of education based on constructivist psychology, focusing on knowledge acquisition rather than general problem-solving skills. Constructivism is the view in psychology that new knowledge is constructed in relation to one’s prior knowledge (Schmidt et al. 2011). This insight has its roots in the work of Piaget and his conception of the mental schema, but was rendered more precise by Anderson from 1977 onwards. Schemata, “frames”, or “scripts” are defined as giving “generic characterizations of things and events”, containing “slots” that can be “instantiated” with particular cases and situations (Anderson et al. 1978, p. 434). This notion influenced the work of Henk Schmidt, who uses Anderson-inspired schema theory to explain what happens during the discussion of a PBL problem (Schmidt 1993), later phrased as activation of a student’s prior knowledge (Schmidt 1983, 1993). The knowledge acquisition view thus incorporates *in a specific way* the context that the information-processing view attempts to leave behind, with the former focusing on learning as guided discovery from a particular vantage point rather than as the learning of abstract general skills.

The Danish model takes it one step further when it comes to contextualisation. This model injects contextualisation by the students, who propose a problem to be addressed, but also contextualization in a more traditional academic through lectures organised in parallel and in line with, or as part of, the PBL course. However, even within the Maastricht and Danish models of PBL the involvement of context is limited to the prior knowledge of students and to the knowledge provided in lectures. Isolation therefore still occurs in these models.

Though the Maastricht and Danish models better embrace the importance of context through activation of prior knowledge or letting students take charge of their learning, both revolve around an overly narrow construal of education as learning, which is meant to produce a particular educational outcome (Biesta 2017). Seeing why and how this is problematic requires looking at PBL from a different standpoint. To this end, in the remaining part of this section we focus on a feature shared across the three models of PBL and point out a parallel problem. PBL, in its focus on a series of separate problems, whether formulated by teachers or students, organises education as a separate activity, only tenuously connected to the world at large. In this sense, education takes place in isolation.

Hannah Arendt, focusing on primary and secondary education, diagnoses a related phenomenon as the “crisis of education” of the mid-twentieth century in the United States (Arendt 1961). She contends that because we are not addressing the world into which our education fits, we are at risk of constructing a completely separate and isolated space for our students. This means that at the end of such an education, students have not been educated to share in the “common sense” of their own society, and thus stand apart from the world. Likewise, in PBL, students can focus on one problem at a time because all three models of PBL are organised around a sequence of separate problems.^1^ While this turns out to improve performance (Schmidt et al. 2009) in the short run, it is arguably also a missed opportunity. What is neglected in these forms of organisation of education is that highly-educated professionals and knowledge workers seldom deal with one isolated problem at a time. It also neglects that the practice of a professional is part of a larger context: a social-ecological system (Sterling 2010). It is important to address these issues by engaging our shared world and its ‘common sense’. These strategies go beyond working efficiently. For example, how should a doctor not only take care of her own patient, but also reflect on her own medical practice from a sustainability point of view? Making such decisions involves specific skills and an additional dimension of reflection in relation to its context: the shared world as a whole. We consider educating for such skills a key requirement of sustainable education. It is here that the aspect of responsibility for a shared world, discussed earlier, enters the stage.

Of course, one could claim this issue is not exclusive to PBL; it is equally a challenge to other forms of education. We do, however, think that *noblesse oblige*. If PBL wants to remain innovative, it should be adjusted such that the larger context is also taken into account. Only in this way can PBL become sustainable.

## Isolation strengthened by a culture of measurement

The two arguments that we have developed thus far are internal to PBL, but the tendency to isolate the process of education is further strengthened by a wider societal factor. The very question of the purpose of education often fades into the background, with criteria of measurement and comparison of educational outcomes. This is clear from (inter)national league tables that compare different pedagogical strategies based on their effectiveness or output. Biesta (2015) goes as far to say that we live in an ‘age of measurement’.

Using objective data to measure pedagogical effectiveness is certainly important because it allows decisions to be based on data rather than beliefs (Biesta 2009). Yet reliance on data is not sufficient by itself. As is well known in the field of educational evaluation, measurement requires evaluation (Biesta 2015; Schwandt and Dahler-Larsen 2006). This requirement of evaluation means we have to decide what we value before we scrutinize data, and only thereafter draw conclusions about what should follow. Measuring the effectiveness of a certain pedagogical approach thus leads far beyond data—the data do not exist separately from the standards we apply to evaluate effectiveness. We first have to decide what data we value. Rather than acting as an incentive for improvement, simply setting technical criteria can elicit responses that are tailored to the criteria while significantly worsening the experience of ‘consumers’ (Biesta 2015; O’Neill 2002).

Further, if measurement relies on evaluation, we should emphasize that the required evaluative judgments are formed within a specific context. The particular “forms of life” (Schwandt and Dahler-Larsen 2006) that inform the evaluation—and are in turn impacted by it—are thus brought into focus. Crucially, this dependence on context and forms of life is missed by contemporary approaches to measurement (Biesta 2015) such that education is incorrectly viewed as an isolated process without reference to context. In this sense, the existing culture of measurement encourages an isolated conception of education, and strengthens education-internal tendencies in the same direction such as the first two critiques we have discussed.

## Sustainable education through acting in/on a shared world

Our critical analysis of PBL in terms of isolation calls for a more thorough consideration of the context that is at stake. One of the original principles of PBL was fostering a connection to society, but it seems somewhat absent from current implementations. Enlarging the scope of this original principle, we argue that it is critical to promote responsibility for a shared world if we wish for PBL to be sustainable. This notion of ‘world’ is rooted in Hannah Arendt’s work and requires some conceptual clarification. We next hope to show the importance of what she calls *action* to education.

Arendt’s work makes a key distinction between three modes that jointly constitute human activity (*vita activa*): labor, work, and action (Voice 2014). Arendt views labor as meeting immediate needs, such as sustaining oneself through nourishment. For that reason, labor has a circular structure and describes mankind as a natural creature: “*animal laborans* who inhabits the earth” (Arendt 1998, p. 140). Work extends labor by defining an activity that is not circular but goal-oriented: it terminates in the fabrication of an object of a certain permanence, such as a table. According to Arendt, mankind as *homo faber* fashions our shared human world (as distinct from the earth) through work. However, the world is the sphere we create not only by fashioning material objects, but also creating institutions—the latter through action. On this level, Arendt situates her aforementioned critique of education in the United States: the crisis she diagnoses stems from the creation of a separate sphere (Arendt 1961), rather than a table at which we may all be seated, or, in our context, a specific, isolated problem to be solved. Our goal in approaching PBL from Arendt’s framework is to overcome the threefold problem of isolation by reconnecting PBL to our shared world.

Action, the third and ‘highest’ sphere of human activity described by Arendt, cannot be defined without engaging in a separate scholarly discussion. For our purposes, it will be sufficient to focus on its importance to education, which has been developed in the work of Biesta (1998, 2017). Action concerns the initiatives of individuals, where what they initiate introduces something new into the world. This capacity for what is new is called “natality” by Arendt, and according to her, “natality is the essence of education” (Arendt 1961, p. 174). Connecting these different strands, within the context of education we define action as articulating the world and creating it anew (Voice 2014). It is important to note that in this definition, as in Arendt’s own framework, action reshapes the common world created through work. In turn, work cannot be conceived without labor and the earth enabling it. In terms of sustainable education, we thus find a series of nested layers that are all integral to the existence of the others, such that cultivating the resilience of each layer becomes an important objective (Sterling 2010).

What does a focus on responsibility for a shared world imply for educational practice? It does not imply that an actual job situation should be replicated in the PBL tutorial; there remains a critical difference between working and studying (Masschelein 2012). However, we should also not throw students in the deep end by asking them to deal with immensely complex situations by themselves. Instead, education should equip students with skills and practical knowledge that will contribute to their common sense and responsibility for a shared world. Reflection on Arendt’s framework provides a direction for the avoidance of the threefold problem of isolation. We propose measures meant to de-emphasize ‘rate of return’ (or other educational outcomes) and to emphasize responsibility for what is held in common. It is also important for this change of focus to be reflected in policy documents on education, so it can guide the (re)development of curricula. Applying this change of focus to the three forms of the problem of isolation criticized above, we can begin to formulate some suggested updates to PBL.

First, the critique of the generalizability of skills should be countered by making context-specific skills more prominent. Education for generalizable skills operates on a level of abstraction that leaves behind the world understood in Arendt’s sense. It is for this reason that the General Problem Solver is out of touch with reality. Only within a specific context do certain skills, and their use, attain relevance. Emphasizing the importance of context delivers us from the illusion (inevitably a disillusion) that students can be trained to become universal knowledge workers. Assigning prominence to context suggests educating in a way that reflects the insights of cognitive psychology and educating within specific (inter)disciplinary domains.

Second, PBL becomes unsustainable when education is organized as a separate activity only tenuously connected to the world at large. This suggests that working with societal partners and real-world problems are both integral to PBL (Jiusto et al. 2013; Wiek et al. 2014). More generally, reflection on how to deal with responsibilities students will face should be part of the program.

Third, in terms of measurement, a long-term perspective should be adopted that de-emphasizes rate of return and emphasizes responsibility for a shared world. Linking education to the world is a vastly different goal than shorter term outcomes such as the acquisition of skills or knowledge. The former approach to education does not specify, for instance, an objective measure that can be used to compare different educational programmes to one another. For these reasons, proposing a focus on responsibility for a shared world implies a break with the wider culture of measurement that is part and parcel of how we approach education today, whether at the level of national policy or local institutions. It thus becomes harder to tell whether a pedagogical approach has been successful upon the graduation of a cohort of students; but, conversely, a higher rate of return is also not a guarantee for success.

## Conclusion

PBL started in the 1960s and has radically changed educational programmes and policies. However, in this paper we have analyzed the issue of isolation within PBL. We think the time has come for an update that considers not only short-term educational outcomes but also education over the long-term: sustainable education. This is all the more pressing because we live in a demanding society, evidenced by high levels of stress and increasing burnout rates (Elflein 2018). PBL has the responsibility to educate students for their futures in a world held in common. This represents a widening of the community orientation originally formulated in PBL—from the local community to the shared world as a whole. We have introduced Arendt’s framework of *vita activa* and made some initial suggestions to update PBL in line with this framework. By adopting and further exploring the implications of responsibility for a shared world, we believe PBL can become more sustainable. Finally, as a further nested stage for sustainable education, we wish to add that this perspective should also be applied to staff (Sterling 2010).

## Footnotes

1 We note that Arendt also associates the crisis of education with the withdrawal of authority from the classroom, at least in relations between adults and children (Arendt 1961, p. 181).
